# Farnesoid X Receptor Activation Enhances Transforming Growth Factor β-Induced Epithelial-Mesenchymal Transition in Hepatocellular Carcinoma Cells

**DOI:** 10.3390/ijms19071898

**Published:** 2018-06-28

**Authors:** Masahiko Kainuma, Ichiro Takada, Makoto Makishima, Keiji Sano

**Affiliations:** 1 Division of Biochemistry, Department of Biomedical Sciences, Nihon University School of Medicine, 30-1 Oyaguchi-kamicho, Itabashi-ku, Tokyo 173-8610, Japan; kainumamasahiko23@yahoo.co.jp; 2 Department of Surgery, Teikyo University School of Medicine, 2-11-1 Kaga, Itabashi-ku, Tokyo 173-8605, Japan; k2sano-tky@umin.ac.jp

**Keywords:** farnesoid X receptor, bile acid, hepatocellular carcinoma, epithelial–mesenchymal transition, guggulsterone, transforming growth factor β, N-cadherin, focal adhesion kinase

## Abstract

Farnesoid X receptor (FXR) is a receptor for bile acids and plays an important role in the regulation of bile acid metabolism in the liver. Although FXR has been shown to affect hepatocarcinogenesis through both direct and indirect mechanisms, potential roles of FXR in epithelial–mesenchymal transition (EMT) in hepatocellular carcinoma (HCC) remain unclear. We examined the effect of several FXR ligands on EMT-related morphological changes in HCC cell lines, such as HuH-7 and Hep3B cells. FXR agonists (chenodeoxycholic acid, GW4064, and obeticholic acid)—but not an antagonist (guggulsterone)—induced actin polymerization and expression of N-cadherin and phosphorylated focal adhesion kinase, although they were less effective than transforming growth factor β (TGF-β). FXR agonist treatment enhanced TGF-β-induced EMT morphologic changes and FXR antagonist inhibited the effect of TGF-β. Thus, FXR activation enhances EMT in HCC and FXR antagonists may be EMT-suppressing drug candidates.

## 1. Introduction

Hepatocellular carcinoma (HCC) is the most common primary cancer of the liver and is the third leading cause of cancer-related death in the world [1]. HCC can be cured by surgical resection, which is available only for patients with early-stage disease. High mortality in HCC patients is due to invasion, intra- and extrahepatic metastasis, and postsurgical recurrence. Epithelial–mesenchymal transition (EMT) plays a role in the early steps of invasion and metastasis in many cancers, including HCC [2]. Therapies targeting EMT could prolong survival of HCC patients.

Farnesoid X receptor (FXR) is a nuclear receptor activated by bile acids and plays an essential role in the regulation of bile acid metabolism by suppressing the synthesis and import of bile acids in hepatocytes and stimulating their biliary excretion [3,4,5,6]. FXR also regulates glucose and lipid metabolism. FXR activation protects hepatocytes against bile acid-induced cytotoxicity, inhibits hepatic lipogenesis, and increases insulin sensitivity [7]. Indeed, the FXR ligand obeticholic acid is being studied in clinical trials for nonalcoholic steatohepatitis and primary biliary cholangitis [8,9].

In addition to metabolic regulation, FXR is involved in hepatic regeneration and carcinogenesis. FXR activation accelerates liver regeneration and induces forkhead box M1 transcription factor, a cell cycle regulator [10,11]. FXR also promotes liver regeneration through induction of fibroblast growth factor 15 in the intestine [12]. On the other hand, spontaneous hepatocarcinogenesis is induced in FXR-null mice [13,14]. Interestingly, hepatocarcinogenesis in FXR-null mice is repressed by intestinal FXR transgene expression [15]. Dysregulation of bile acid metabolism in the intestine of FXR-null mice is suggested to influence hepatocarcinogenesis. Thus, FXR influences hepatocarcinogenesis through both direct and indirect mechanisms. In this study, we report that FXR activation enhances EMT of HCC cells.

## 2. Results

### 2.1. FXR Agonist Induces EMT Phenotypes in HCC Cells

**Figure 1:**
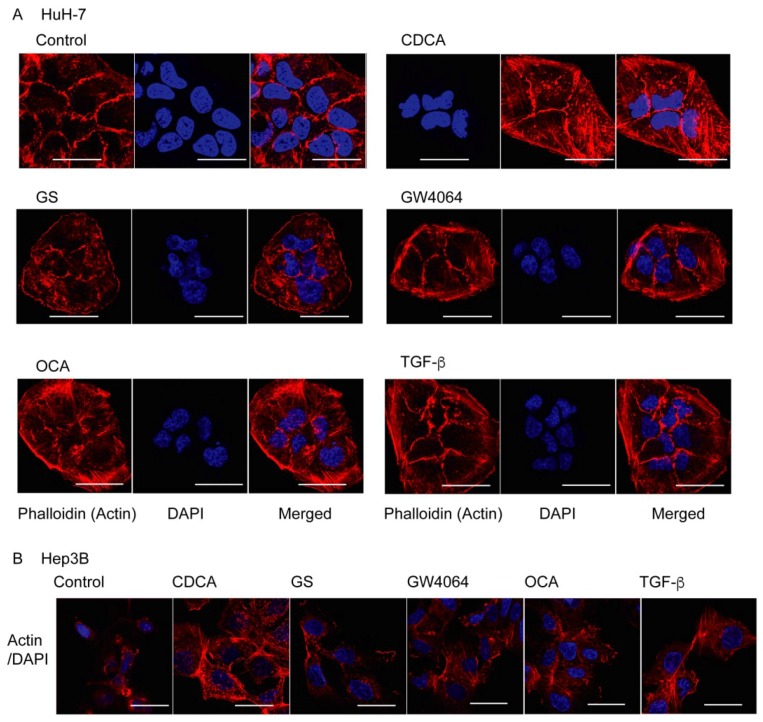
Farnesoid X receptor (FXR) activation induces actin polymerization in HuH-7 cells (**A**) and Hep3B cells (**B**). Cells were treated with vehicle control (Control), 100 μM chenodeoxycholic acid (CDCA), 32 μM guggulsterone (GS), 10 μM GW4064, 10 μM obeticholic acid (OCA), or 10 ng/mL transforming growth factor β (TGF-β) for 48 h and stained with rhodamine phalloidin (**red**) to detect actin polymerization and with DAPI (**blue**) to show nuclei. Scale bar, 50 μm.

To examine whether FXR regulates EMT in HCC cells, we treated HuH-7 cells with FXR ligand for 48 h and assessed morphological changes. Chenodeoxycholic acid (CDCA) is a potent natural FXR agonist [3] and GW4064 and obeticholic acid (OCA) are synthetic agonists [16,17], while guggulsterone (GS) is an FXR antagonist that has been identified as the cholesterol-lowering agent in the extract of the guggul tree [18]. Similar to transforming growth factor β (TGF-β), which is a strong EMT inducer for HCC cells [2], the FXR agonists GW4064, CDCA, and OCA, but not the antagonist GS, enhanced actin polymerization in HuH-7 cells (Figure 1A). Increased actin polymerization was also observed in Hep3B cells treated with CDCA, GW4064, OCA, and TGF-β, but not in GS-treated cells (Figure 1B).

**Figure 2:**
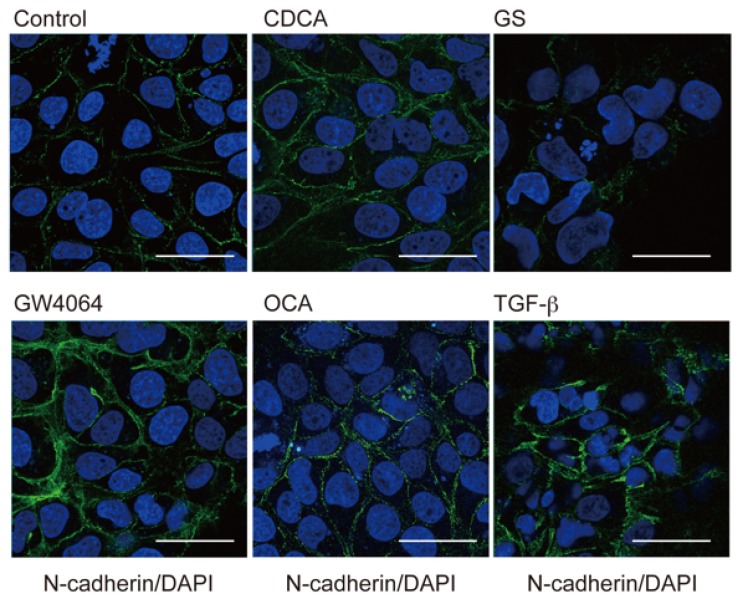
FXR activation induces N-cadherin expression in HuH-7 cells. Cells were treated with vehicle control (Cont), 100 μM CDCA, 32 μM GS, 10 μM GW4064, 10 μM OCA, or 10 ng/mL TGF-β for 48 hours and stained for N-cadherin (**green**) and DAPI (**blue**). Scale bar, 50 μm.

**Figure A1:**
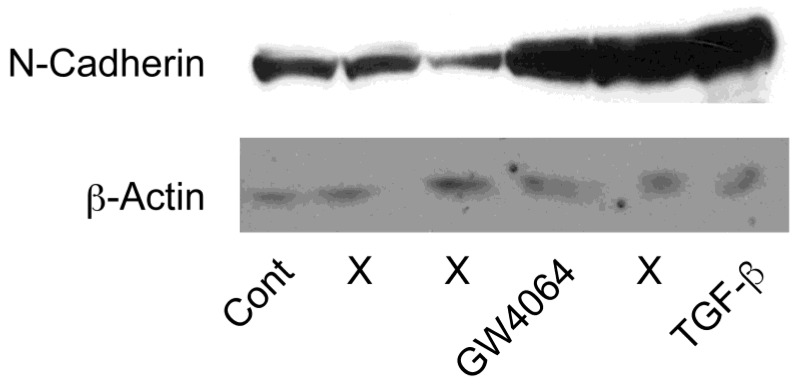
Western blotting for N-cadherin. Cells were treated with vehicle control (Cont), 10 μM GW4064, or 10 ng/mL TGF-β for 48 h. Lanes indicated with an X were not subjected for evaluation.

We also examined the expression of another EMT marker, N-cadherin, in HuH-7 cells [2]. In agreement with a previous report [19], TGF-β treatment induced N-cadherin expression (Figure 2). Immunostaining also showed increased N-cadherin expression in cells treated with GW4064, CDCA, and OCA, but not with GS. In a Western blot analysis, increased N-cadherin protein levels were also observed in cells treated with GW4064 (Figure A1).

**Figure 3:**
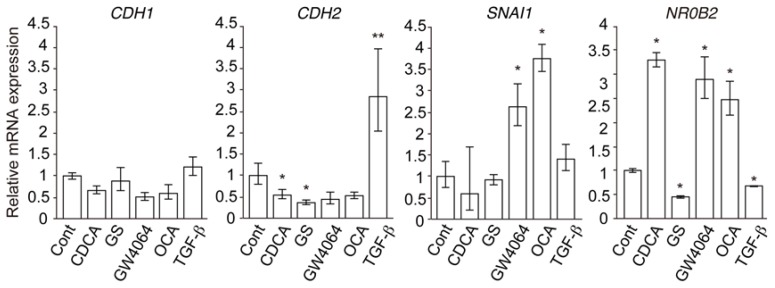
Expression of epithelial–mesenchymal transition (EMT)-related genes and the FXR target gene *NR0B2* in HuH-7 cells. Cells were treated with vehicle control (Cont), 100 μM CDCA, 32 μM GS, 10 μM GW4064, 10 μM OCA, or 10 ng/mL TGF-β for 48 h. * *p* < 0.05; ** *p* < 0.01 versus Cont.

Next, we examined mRNA expression of EMT-related genes. Although TGF-β increased mRNA expression of *CDH2*, which encodes N-cadherin, GW4064 and OCA had no effect and CDCA and GS decreased mRNA levels in HuH-7 cells (Figure 3). GW4064 and OCA increased *SNAI1* mRNA levels but CDCA, GS, and TGF-β did not. Expression of *CDH1*, a gene encoding E-cadherin, was not changed in any cell conditions. Expression of the FXR target gene *NR0B2*, which encodes the small heterodimer partner [20], was increased by treatment with CDCA, GW4064, and OCA, while it was slightly decreased in cells treated with GS and TGF-β. These findings indicate that FXR agonists induce EMT phenotypes in HCC cells in a slightly different manner to TGF-β.

### 2.2. Combined Effect of FXR Ligand and TGF-β in EMT of HCC Cells

**Figure 4:**
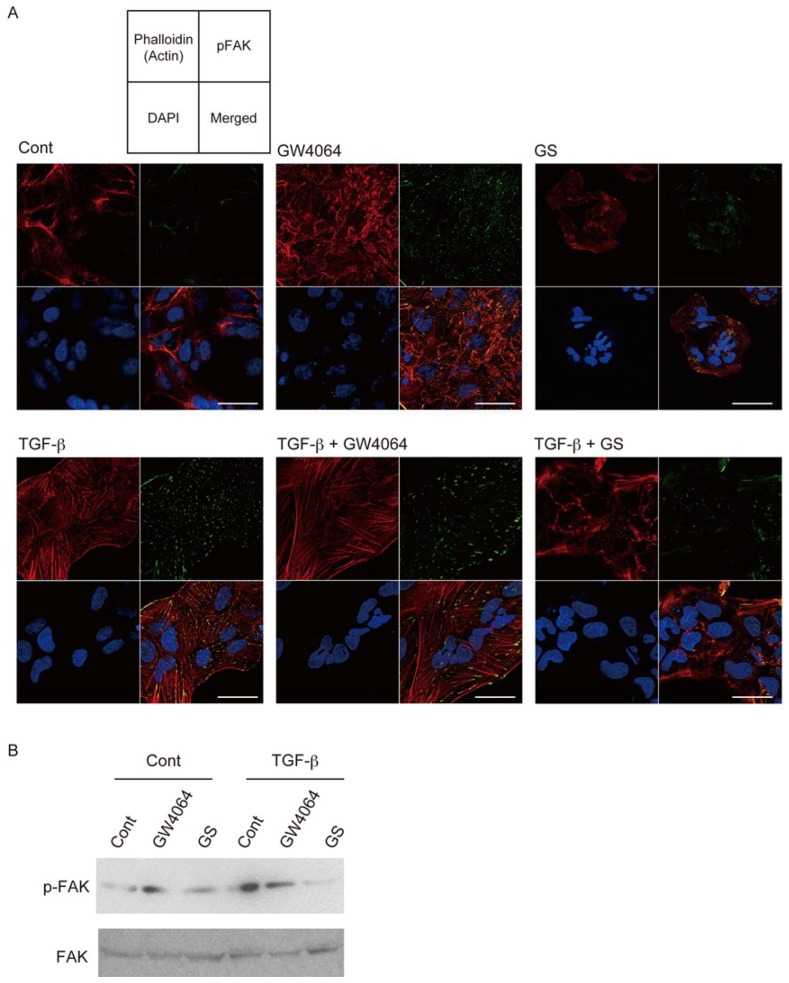
Combined effects of TGF-β and GW4064 or GS on phosphorylated FAK (p-FAK) expression in HuH-7 cells. (**A**) Actin polymerization (**red**) and phosphorylated FAK (p-FAK; **green**); DAPI (**blue**); and (**B**) Western blotting for p-FAK and total FAK. Cells were treated with vehicle control (Cont), 10 μM GW4064, or 32 μM GS in the absence or presence of 10 ng/mL TGF-β for 48 h. Scale bar, 50 μm.

Treatment of HuH-7 cells with TGF-β or GW4064 induced expression of phosphorylated focal adhesion kinase (FAK), a marker correlated with invasion activity of HCC [21] (Figure 4A). Phosphorylated FAK co-localized with polymerized actin and expression patterns of these proteins were different in HuH-7 cells treated with TGF-β and those with GW4064. TGF-β induced mesenchymal morphological changes more effectively than GW4064. GS did not induce these findings. Western blotting showed that GW4064 and TGF-β increased phosphorylated FAK expression (Figure 4B). TGF-β plus GW4064 did not further increase its expression. Interestingly, GS suppressed the EMT phenotype induced by TGF-β (Figure 4A,B).

**Figure 5:**
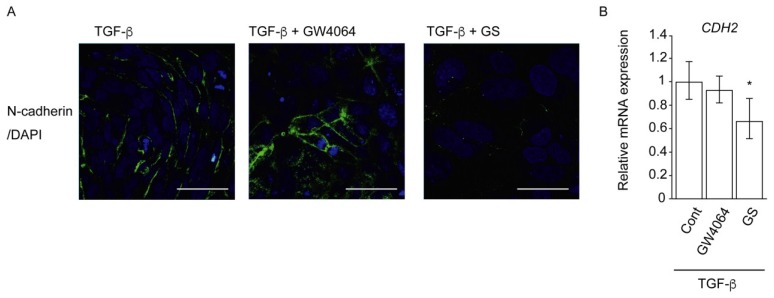
Combined effects of TGF-β and GW4064 or GS on N-cadherin expression in HuH-7 cells. (**A**) N-cadherin protein expression (**green**); and (**B**) *CDH2* mRNA expression. Cells were treated with vehicle control (Cont), 10 μM GW4064, or 32 μM GS in the absence or presence of 10 ng/mL TGF-β for 48 h. Scale bar, 50 μm. * *p* < 0.05 versus Cont.

The addition of GW4064 increased and that of GS suppressed TGF-β-induced N-cadherin expression in HuH-7 cells (Figure 5A). In contrast to the immunostaining findings, GW4064 did not change and GS suppressed *CDH2* mRNA expression in TGF-β-treated cells (Figure 5B).

## 3. Discussion

In this study, we found that FXR agonism promotes and FXR antagonism suppresses EMT phenotypes in HCC cells. In contrast, antitumor effects of FXR agonists have been reported. FXR agonist treatment inhibits proliferation of SK-GI-18 cells, which are FXR-overexpressing SK-Hep-1 cells, and suppresses tumor growth and metastasis in an orthotopic xenograft model with these cells in nude mice [22]. Recently, OCA was reported to suppress proliferation, migration, and invasion of HepG2 cells and HuH-7 cells [23]. Our preliminary experiments showed higher concentrations of CDCA, GS, GW4064, and OCA inhibited cell proliferation. To avoid their toxic effects, we chose non-toxic concentrations of these compounds (100 μM CDCA, 32 μM GS, 10 μM GW4064, and 10 μM OCA). Effective concentrations of these compounds are dependent on cell culture conditions, such as cell density, medium, serum, and duration. The discrepancy between our results and others may be due to the concentrations of ligands or activation status of FXR. Super-physiological FXR activation may suppress proliferation and migration/invasion of HCC. Our results suggest that physiological FXR activation promotes EMT phenotypes. Hepatocarcinogenesis is enhanced in FXR-null mice [13,14] and FXR expression is decreased in human HCC samples [24]. These findings support the tumor-suppressing role of FXR. On the other hand, there is a significant association between nuclear FXR expression and Ki-67 labeling in human HCC samples [25]. FXR activation suppresses inflammatory responses [6]. FXR may act as a tumor suppressor at the initiation or early stage of HCC through the regulation of bile acid metabolism and inflammation and play a different role in the late stage of HCC as an EMT enhancer.

Our results showed that FXR agonists were less effective than TGF-β in EMT morphology induction (Figure 1, Figure 2 and Figure 4). TGF-β, but not FXR agonists, effectively induced *CDH2* expression (Figure 3). On the other hand, GW4064 and OCA, but not TGF-β, increased *SNAI1* expression (Figure 3). GS treatment decreased *CDH2* expression induced by TGF-β (Figure 5). These findings suggest that FXR agonists enhance EMT phenotypes in a manner different from TGF-β. We could not find an FXR-responsive element in the *CDH1*, *CDH2*, and *SNAI1* promoters. Similar to the EMT-suppressing effect in our results (Figure 4 and Figure 5), GS decreases motility and invasion of pancreatic cancer cells [26]. GW4064 enhances and GS inhibits EMT changes in TGF-β-treated human bronchial epithelial cells [27], and bile acids, including CDCA, also induce EMT in human lung alveolar cells [28]. Interestingly, lithocholic acid, which is a weak FXR agonist [3], stimulates TGF-β release from lung fibroblasts [28]. FXR may enhance EMT by modulating TGF-β signaling. There was a discrepancy between protein and mRNA expression of N-cadherin (Figure 2 and Figure 3). FXR agonists stimulate insulin secretion in mouse pancreatic β cells via an FXR-mediated non-genomic action [29]. It remains unclear whether the effect of FXR ligand on EMT is mediated by a genomic or non-genomic action. Further studies are needed to determine the underlying mechanism of FXR action on EMT.

FXR activation inhibits hepatic inflammation [6]. Recently, OCA has been studied in clinical trials for nonalcoholic steatohepatitis and primary biliary cholangitis [8,9]. On the other hand, disturbance in bile acid homeostasis, including accumulation of FXR-activating bile acids, is associated with nonalcoholic fatty liver and nonalcoholic steatohepatitis [30]. Increasing levels of deoxycholic acid, which is another weak FXR agonist [3], promote hepatocellular carcinogenesis [31]. OCA enhanced TGF-β-induced EMT phenotypes (Figure 4 and Figure 5). Although FXR plays a role in protection against bile acid toxicity and pathogenesis by regulating bile acid metabolism [6], OCA and other FXR agonists should be used with caution for patients with HCC. The FXR antagonist GS exhibits cholesterol-lowering activity [18]. Therefore, GS or synthetic FXR antagonists [32] may be able to prolong survival of late-stage HCC patients.

## 4. Materials and Methods

### 4.1. Cell Culture

Human HCC HuH-7 cells and Hep3G cells were obtained from the American Type Culture Collection and cultured in DMEM high glucose (Wako Pure Chemical Industries, Osaka, Japan) supplemented with 10% fetal bovine serum, 50 U/L penicillin, and 50 μg/L streptomycin. Cell were treated with vehicle control, 100 μM CDCA (Wako Pure Chemical Industries), 32 μM GS (Enzo Life Science, Farmingdale, NY, USA), 10 μM GW4064 (ChemScene, Monmouth Junction, NJ, USA), 10 μM OCA (AdipoGen Life Sciences, San Diego, CA, USA) and/or 10 ng/mL TGF-β (PeproTech, Rocky Hill, NJ, USA) for 48 h.

### 4.2. Immunolostaing

Cells were fixed in 4% paraformaldehyde and blocked in 5% skim milk/phosphate buffer saline with 0.1% Tween-20 (PBST). After washing in PBST, cell samples were incubated with rabbit anti-FAK(p397) antibody (Abcam, Cambridge, UK) or mouse anti-N-cadherin antibody (BD Biosciences, San Jose, CA, USA) in 5% skim milk/PBST, washed with PBST twice, and incubated with Akexa488-conjugated anti-rabbit IgG antibody or Alexa546-conjugated anti-mouse IgG antibody (Thermo Fisher Scientific, Waltham, MA, USA). Cell specimens were also stained with rhodamine phalloidin (Cytoskelton, Inc., Denver, CO, USA). Mounted cell specimens were analyzed with a confocal microscope (ZSM710; Carl Zeiss, Jena, Germany).

### 4.3. Western Blotting

Proteins were subjected to sodium dodecyl sulfate-polyacrylamide gel electrophoresis and transferred to nitrocellulose membranes. Membranes were blocked with PBST with 2.5% skim milk, incubated with anti-FAK(p397) antibody (Abcam), anti-FAK antibody (BD Biosciences), anti-N-cadherin antibody (BD Biosciences), or anti-β-actin antibody (Sigma-Aldrich, St. Louis, MO, USA), and then with horseradish peroxidase-coupled secondary antibody (Agilent, Santa Clara, CA, USA), and visualized with the ECL Western Blotting Detection Reagents (GE Healthcare, Chalfont St. Giles, UK).

### 4.4. mRNA Expression

Total RNA was extracted using TRIZOL (Thermo Fisher Scientific) and cDNA was synthesized using PrimeScript Reverse Transcriptase (Takara Bio, Otsu, Japan). Quantitative polymerase chain reaction was performed using ABI PRISM7000 (Thermo Fisher Scientific) with Light Cycler SYBR Green I Master Mix (Takara Bio), and quantification was performed as reported previously [33]. Used primer sequences were: *CDH2*, 5’-TGG AGA CAT TGG GGA CTT CA-3’ and 5’-ATT AAG GGA GCT CAA GGA CC-3’; *CDH1*, 5’- GAA GGT GAC AGA GCC TCT GGA TAG-3’ and 5’-CTG GAA GAG CAC CTT CCA TGA-3’; *SNAI1*, 5’-AAG ATG CAC ATC CGA AGC CA-3’ and 5’-CTT GAC ATC TGA GTG GGT CT-3’; *NR0B2*, 5’-AAT ATG CCT GCC TGA AAG GGA-3’ and 5’- GAT AGG GCG AAA GAA GAG GTC C-3’; *GAPDH*, 5’-ACT TCG CTC AGA CAC CAT GG-3’ and 5’-GTA GTT GAG GTC AAT GAA GGG-3’. The mRNA levels were adjusted to those of *GAPDH*, a gene encoding glyceraldehyde-3-phosphate dehydrogenase.

### 4.5. Statistical Analysis

Data are presented as the mean ± S.D. We performed one-way ANOVA followed by Tukey’s multiple comparisons to assess significant differences.

## Abbreviations

HCC	Hepatocellular carcinoma
EMT	Epithelial-mesenchymal transition
FXR	Farnesoid X receptor
CDCA	Chenodeoxycholic acid
OCA	Obeticholic acid
GS	Guggulsterone
TGF-β	Transforming growth factor β
FAK	Focal adhesion kinase
PBST	Phosphate buffer saline with Tween-20
